# Prevalence and determinants of active trachoma among preschool-aged children in Dembia District, Northwest Ethiopia

**DOI:** 10.1186/s40249-017-0345-8

**Published:** 2017-10-09

**Authors:** Ayanaw Tsega Ferede, Abel Fekadu Dadi, Amare Tariku, Akilew Awoke Adane

**Affiliations:** 1 Department of Optometry, School of Medicine, College of Medicine and Health Sciences, University of Gondar, Gondar, Ethiopia; 2 Department of Epidemiology and Biostatistics, Institute of Public Health, College of Medicine and Health Sciences, University of Gondar, Gondar, Ethiopia; 3 Department of Human Nutrition, Institute of Public Health, College of Medicine and Health Sciences, University of Gondar, Gondar, Ethiopia

**Keywords:** Active trachoma, Cross-sectional study, Preschool- aged children, northwest Ethiopia

## Abstract

**Background:**

Trachoma is an infectious eye disease caused by *Chlamydia trachomatis*, which is the leading infectious cause of blindness worldwide. In areas where trachoma is endemic, active trachoma is common among preschool-aged children, with varying magnitude. This study aimed to estimate the prevalence of active trachoma and associated risk factors among preschool-aged children in Dembia District, northwest Ethiopia.

**Methods:**

A community-based cross-sectional survey was conducted among preschool-aged children of northwest Ethiopia. Multistage systematic random sampling was used to select 695 subjects. Trained clinical optometrists subjected each child to an ocular examination and assessed the presence of active trachoma. Face to face interview using pretested and structured questionnaire were conducted to collect data on possible risk factors. Trachoma cases were graded following a World Health Organization simplified grading scheme. All statistical analysis was carried out using the SPSS software version 20. Adjusted odds ratios (a*OR*s) with 95% confidence intervals (*CI*s) were used to identify factors associated with active trachoma.

**Results:**

Of the 681 preschool-aged children studied, 18% (95% *CI*: 15.4% – 21.1%) had a prevalence of active trachoma. Children who had clean faces (absence of nasal and ocular discharges) had a lower chance of having active trachoma [a*OR* = 0.55, 95% *CI*: 0.37 – 0.82]. The odds of having active trachoma decreased with an increase in the distance to a water point [a*OR* = 0.51, 95% *CI*: 0.33 – 0.78]. Similarly, no or poor utilization of liquid waste disposal in the child’s household was associated with an increased chance of having active trachoma [a*OR* = 3.83, 95% *CI*: 1.26 – 11.61].

**Conclusion:**

The prevalence of active trachoma in these preschool-aged children was found to be high and needs special interventions that focus on educating families about proper face washing, liquid waste disposal, and improving safe water supply near the households.

**Electronic supplementary material:**

The online version of this article (doi:10.1186/s40249-017-0345-8) contains supplementary material, which is available to authorized users.

## Multilingual abstracts

Please see Additional file 1 for translations of the abstract into the five official working languages of the United Nations.

## Background

Trachoma is an infectious eye disease caused by the bacterium *Chlamydia trachomatis*. It is the leading infectious cause of blindness worldwide [1–3]. Children who have the active stages of the disease are the reservoir of infection [4]. Recurrent episodes of infection and the associated chronic conjunctival inflammation initiate a scarring process that ultimately leads to irreversible blindness [5, 6].

According to the World Health Organization (WHO), 1.9 million people worldwide are blind or visually impaired due to trachoma [7]. In areas where trachoma is endemic, active trachoma is common among preschool-aged children, with prevalence rates that can be as high as 60 – 90% [4, 6–9]. Globally, more than 200 million people live in trachoma endemic areas, 12.4 million children are suffering from active trachoma and Africa is the most affected continent with 27.8 million (68.5%) children affected. Nearly 50% of the global burden of active trachoma is highly distributed in three countries: Ethiopia, Malawi, and Nigeria [10]. Global loss of productivity related to impaired vision and blindness from trachoma is thought to be as high as $US 5.3 billion annually [11].

The risk factors for trachoma vary between settings depending on individual, economic, and environmental factors. Studies from some parts of Africa reported that children with trachoma were more likely to have ocular and nasal discharge [11–13]. Unclean faces, or clean faces but with flies, time to fetch water, overcrowding, garbage within the compound, children aged 3–5 years, less frequent face washing, practice of open defecation, household cattle ownership, high household fly density, and long distance to the nearest water source are among the factors that have been associated with active trachoma [11, 14–17].

In Ethiopia, trachoma remains a major public health problem [18–20]. The Ethiopian National Survey on Blindness, Low Vision, and Trachoma, carried out from 2005 to 2006 estimated that the national prevalence of active trachoma was 40.14%, with some regional variation; the highest prevalence being in the Amhara region (62.60%) [18]. The prevalence rate of active trachoma in various districts in Ethiopia were; 22% in Goro zone, 22.8% in Gurage zone, and 24.10% in east Gojjam zone [17, 19, 20].

Since the prevalence and risk factors associated with trachoma varies from setting to setting, knowing the magnitude and risk factors associated with trachoma in this specific community would help to understand the disease magnitude and transmission. This study was conducted after successive mass drug administration was carried out in the area and could be considered as a preliminary assessment after the intervention. Results from the study could thus help make the intervention more focused and effective.

## Materials

### Study setting and design

This community based cross-sectional study was conducted in the Dembia District, which is located in the northwest part of Ethiopia from January to February 2015. This district has 45 kebeles *(smallest administration unit),* of which 40 are rural kebeles. This district has a population of 315,903, with a 260.1/km^2^ population density. Out of the total population, preschoolers (grade 1 – 4) comprise 5.7% (18006) [21]. There are 10 health centers and 40 health posts in the district. All preschool-aged children living in the district for at least 6 months were included in the study.

### Sample size and sampling procedure

The minimum sample size was 695 preschool-aged children, which was determined using a single population proportion formula [*n* = [(Z_α/2_)^2^*P (1-P)]/d^2^], by assuming a 95% confidence level of Z α/_2_ = 1.96, a margin of error of 4%, a design effect of 2, a prevalence of trachoma among preschool-aged children as 15.6% based on similar studies conducted nearby [22] and accounting for a 10% non-response rate.

Multi-stage random sampling followed by systematic random sampling was employed to reach the study subjects. Initially, nine kebeles (one urban and eight rural) were selected randomly using the lottery method. A total number of eligible preschool-aged children residing in the selected kebele’s were obtained from the local administration. The final sample size was proportionally allocated to each kebeles based on the total number of preschool-aged children living in each kebeles. Systematic random sampling with a K interval of 2 (K was determined by dividing the total number of households which had a preschool-aged child in the kebele by the proportion of sample size allocated for that specific kebele) was used to select households. If more than one preschool-aged child lived in the same household, one child was selected randomly using the lottery method. When mother-child pairs were not available at the time of the data collection, two repeated visits were made, and if the same situation occurred the next household was then considered.

### Data collection

A structured questionnaire was used to collect the socio-demographic and economic characteristics, health-related characteristics, and trachoma-related characteristics of the participant. Language experts translated the questionnaire from English to Amharic and back to English to ensure consistency. Researchers conducted pretests on an unselected district population by taking 10% of the total sample size and used the revised questionnaire for final data collection. Two public health experts and 12-trained data collectors (two clinical optometrists and 10 clinical nurses) were recruited for supervision and data collection, respectively. The investigators (ATF, AFD, AT, AAA) coordinated the overall activities of data collection.

A household wealth index was computed using the composite indicator for urban and rural residents using assets: livestock ownership, selected household assets (TV, furniture), size of agricultural land and quantity of crop production. Principal component analysis was performed to categorize the households’ wealth index into lowest, middle, and highest. Having a ‘clean face’ was assessed as an absence of nasal and ocular discharges on the child’s face during data collection time only.

### Clinical assessment of trachoma

Following the face-to-face data collection interview, trained clinical optometrists examined each child by ocular examination. The clinical optometrists performed a detailed ophthalmic examination with strict adherence to standard methods and procedures. Optometrists used an ophthalmoscope, pentorch, and binocular loupe that has 2.5× magnifying power to identify clinical signs of trachoma: trachomatous inflamation-intense (TI), trachomatous inflammation-follicular (TF), trachomatous conjunctival scar (TS), trachomatous trichiasis (TT), and corneal opacity (CO). Eyelid eversion (turning out) was done using an aseptic technique using cotton tip applicators and alcohol used for hand disinfection.

### Data analysis

The software Epidemiological Information (Epi-Info) version 3.5.1 was used for data entry while Statistical Package for Social Sciences (SPSS) version 20 was used for analysis. Descriptive statistics such as frequency and cross tabulation were calculated for selected variables. A backward binary logistic regression model was used to identify factors associated with active trachoma. Both crude odds ratio (c*OR*) and adjusted odds ratio (a*OR*) with 95% confidence intervals (*CI*) were used to show the association between active trachoma and selected variables. In addition a*OR* with their 95% *CI*s were used to identify significantly associated factors. The model’s fitness test was evaluated using the Hosmer _ Lemeshow goodness of fit test.

### Ethical clearance

Ethical clearance was obtained from the Institutional Review Board of the University of Gondar. Permission to conduct the research was obtained from the Amhara regional health bureaus and respective health offices. Written consent was obtained from the participants after explaining the purpose of the study to them. To ensure confidentiality, their names and other personal identifiers were not registered in the format. Preschool-aged children who were found to have a sign of active trachoma were provided with 0.1% topical tetracycline and those with signs of advanced disease (TS/TT/CO) were referred to the ophthalmic specialty clinic at the Gondar University Referral Hospital.

## Results

### Socio-demographic characteristics

Six hundred and eighty-one children (681) were interviewed giving a response rate of 98% (all were interviewed and examined). The mean age (± *SD*) of the children was 41.58 (± 11.27) months and slightly more than half (53.6%) were boys. The majority of the children’s mothers were housewives (95.4%), married (90.0%), and unable to read or write (77.1%) (Table 1).

**Table 1:** Socio-demographic characteristics of the study participants in Dembia District, northwest Ethiopia, 2015

Variables	Frequency	Percent
*Age in months*		
24 – 36	288	42.3
37 – 48	233	34.2
≥ 49	160	23.5
*Sex*		
Male	365	53.6
Female	316	46.4
*Residence*		
Urban	47	6.9
Rural	634	93.1
*Marital status of the mother*		
Single	25	3.7
Married	613	90
Divorced	43	6.3
*Number of children aged below five in the household*		
One	433	63.6
Two and above	248	36.4
*Mother’s education level*		
Uneducated	525	77.1
Primary	58	8.5
Secondary and above	98	14.4
*Mother’s occupation*		
Housewife	650	95.4
Merchant	31	4.6
*Mother’s age*		
15 – 34	495	72.7
35 – 48	186	27.3
*Father’s education level*		
unable to read or write	410	60.2
Primary complete	177	26
Secondary and above	94	13.8
*Wealth index*		
Low	226	33.2
Medium	228	33.5
High	227	33.3
*Children usually stay at..*		
Home	472	69.3
Out of home	209	30.7

### Health and environmental related characteristics

Four hundred and eighty mothers (70.5%) reported that they had given birth at home. More than 80% of the respondents lacked proximate access to a water supply. Around half the respondents lacked household solid (49.9%) and liquid waste disposal (51.8%) facilities. Almost all of the mothers (93.4%) said that their kids had azithromycin treatment as part of trachoma elimination efforts prior to the survey. According to the mothers’ reports about their children, 49% (330/681) of the children washed their faces two times a day and 54.9% (374/681) of the children had clean face (Table 2).

**Table 2:** Health and environmental-related characteristics of study participants in Dembia District, northwest Ethiopia, 2015

Variables	Frequency	Percent
*Place where child was delivered*		
Home	480	70.5
Health facility	201	29.5
*Initiation of breastfeeding*		
Early initiation	479	70.3
Late initiation	202	29.7
*Access to treated water supply*		
Yes	118	17.3
No	563	82.7
*Daily water consumption of the household*		
≤ 40 l	267	39.2
41 – 60 l	253	37.2
≥ 61	161	23.6
*Time required to fetch water*		
≤ 15 min	262	38.5
16 – 30 min	330	48.5
> 30 min	89	13.1
*Availability of a solid waste disposal facility*		
Yes	341	50.1
No	340	49.9
*Availability of a liquid waste disposal facility*		
Yes	328	48.2
No	353	51.8
*Latrine availability*		
Yes	531	78.0
No	150	22.0
*Latrine functionality*		
Yes	510	74.9
No	21	3.1
Don’t have a latrine	150	22.0
*Main energy source*		
Wood	457	67.1
Electric	21	3.1
Animal dung	203	29.8
*Child had at least one dose of azithromycin in the year preceding*		
Yes	636	93.4
No	45	6.6
*Frequency of face washing in a day (self-reported)*		
Once	175	25.7
Twice	334	49.0
Three times	172	25.3
*Use of soap when washing face (self-reported)*		
Yes	457	67.1
No	224	32.9
*Face condition*		
Clean(no nasal/ocular discharge)	374	54.9
Unclean	307	45.1

### Prevalence of trachoma and associated factors

The prevalence of active trachoma (TI or TF) among preschool-aged children was found to be 18.2% (95% *CI*: 15.4% – 21.1%). Of all the children examined, 37 (29.8%) had clinical signs of TI, while 84 (67.8%) had TF. Each of the other three grades, namely TT, TS, and CO had similar (0.01%) figures. The prevalence of active trachoma was slightly higher among boys (20.8%) than among girls (15.2%).

After adjusting for socio-demographic, economic, child condition, and environmental related factors, the multivariable logistic regression analysis identified face condition, time to fetch water, and utilization of liquid waste disposal facilities as independent predictors for these pre-school- aged children to have active trachoma.

Accordingly, the odds of having active trachoma were 0.55 [a*OR* = 0.55, 95% *CI*: 0.37 – 0.82] times lower among children who had a clean face. Likewise, the odds of having active trachoma were 3.83 [a*OR* = 3.83, 95% *CI*: 1.26 – 11.61] times higher among children from households that had no or an inadequate liquid disposal facility. The odds of having active trachoma were also 0.51[a*OR* = 0.51, 95% *CI*: 0.33 – 0.78] times lower among children whose families fetched water from 16 to 30 min walking distance away from their homes (Table 3).

**Table 3:** Factors associated with active trachoma (TF) or (TI) among preschool-aged children in Dembia District, northwest Ethiopia, 2015

Variables	Active trachoma(TF/TI)		c*OR*,95% *CI*	a*OR*,95% *CI*
	Yes	No		
*Latrine Availability*				
Yes	88	443	1	
No	36	114	1.59(1.03,2.47)	
*Frequency of face washing in a day (self-reported)*				
Once	41	134	1.64(0.96,2.82)	
Twice	56	278	1.08(0.66,1.79)	
Three times	27	145	1	
*Face condition*				
Clean	54	320	0.57(0.39,0.85)	0.55(0.37,0.82)*
Unclean	70	237	1	1
*Number of children aged below five in the household*				
One	70	363	0.69(0.466,1.02)	
Two and above	54	194	1	
*Time required to fetch water*				
≤ 15 min	61	201	1	1
16 – 30 min	45	285	0.52(0.34,0.79)	0.51(0.33,0.78)*
> 30 min	18	71	0.83(0.46,1.51)	0.88(0.47,1.60)
*Children usually stay at*				
Home	93	379	1	
Out of home	31	178	0.71(0.45,1.11)	
*Latrine functionality*				
Yes	84	426	1	
No	4	17	1.19(0.39,3.63)	
Don’t have a latrine	36	114	1.60(1.03,2.49)	
*Use liquid waste disposal hole*				
Yes	50	143	0.93(0.62,1.38)	0.92(0.61,1.38)
No	6	8	3.37(1.13,10.04)	3.83(1.26,11.61)*
Don’t have liquid waste disposal facilities	68	306	1	1

*P-value <0.05

*Hosmer Lemeshow goodness of fit test was determined as Chi-square of 1.74 with a *P*-value of 0.9

## Discussion

Trachoma remains a major cause of avoidable blindness among underprivileged populations in many areas of Africa including Ethiopia. The problem is severe if not detected and treated as early as possible in preschool-aged children. The prevalence of active trachoma TI and/or TF in these preschool-aged children was found to be 18.2%, which is higher than the WHO recommendation. This study finding is higher compared to result from studies conducted among preschool-aged children in Sao Paulo Brazil [23], and Gambia [11] and lower than in studies conducted in Tanzania [11], Egypt [24] and northeastern Nigeria. The variation in prevalence might be attributed to the differences in the year of study, socio-demographic factors, study settings, and the ages of the study population.

In agreement with previous evidence [23–25], an unclean face is significantly associated with higher prevalence of active trachoma. The possible explanation could be that unclean faces can attract flies. The female eye-seeking fly, of the species Musca sorbens, is a possible mechanical vector of *Chlamydia trachomatis*. In addition, nasal or ocular discharge may result in an unclean face, which has been found to be associated with active trachoma in different setting (27-29). The infected discharge would aid the transmission of active trachoma through fingers, fomites, or flies.

Similarly, in this study the odds of trachoma among in children whose households do not utilize a liquid waste disposal pit was almost four times that of children whose households properly use a liquid waste disposal facility. Studies that has been conducted elsewhere [12, 13, 26] reported similar findings. A reason for this is that an uncontrolled waste disposal practice will result in a favorable condition for flies to breed.

Time to fetch water is also an important factor that affects the occurrence of trachoma in preschool-aged children. Travelling a long distance to fetch water has been associated with trachoma in several others studies [26–28]. On the contrary, the current study depicts an inverse relationship between the time to fetch water and trachoma morbidity. The time required to fetch water might vary between individuals who fetch water. For rural communities, access to safe water would be more practical if assessed using distance. The other possible explanation might be that the water that is fetched and used by the family might not be safe enough for face washing purpose. Having adequate water might not be a guarantee for its quality [29].

The limitation of this study lies in the difficulty to establish a temporal relationship between trachoma morbidity and its risk factors. This study is also subject to residual confounding since some potential factors such as fly density, distance to a water source, and water quality issues were not well addressed or measured.

## Conclusions

The prevalence of active trachoma in the preschool-aged children studied in Dembia District, northwest Ethiopia was found to be high and it is much higher than the WHO recommendation. Children who had unclean faces and those from households with poor utilization of a liquid waste disposal facilities were more likely to be affected by active trachoma. Intervention modalities that would address the identified risk factors are highly recommended to prevent and control active trachoma in this setting.

## Additional file

Additional file 1: Multilingual abstracts in the five official working languages of the United Nations. (PDF 624 kb)

## Abbreviations

a*OR*: Adjusted odds ratio
*CI*: Confidence interval
SPSS: Statistical package for social sciences
TF: Trachomatous inflammation – follicular
TI: Trachomatous inflammation- intence
TS: Trachomatous conjuctival scar
TT: Trachomatous trichiasis
WHO: World Health Organization
