# Supplementary material for: Prevalence and determinants of active trachoma among preschool-aged children in Dembia District, Northwest Ethiopia
Source: Infect Dis Poverty. 2017 Oct 9;6:128. doi: 10.1186/s40249-017-0345-8 (PMC5632823; doi:10.1186/s40249-017-0345-8)
Supplement: Additional file 1: — Multilingual abstracts in the five official working languages of the United Nations. (PDF 624 kb) [file 40249_2017_345_MOESM1_ESM.pdf]

Translation of the abstract into the five official working languages of the United Nations

نسبة إنتشار التراخوما النشطة والعوامل المحددة لها بين الأطفال في سن ما قبل المدرسة في منطقة ديمبيا بشمال غرب إثيوبيا

آيناو تسيجا فريدريه, أبيل فيكادو دادي, أماري تاريكو, أكيليو أووكي أداني

الملخص
الخلفية: التراخوما هي مرض معدي للعين تسببه المتدثرة الحثرية ويعتبر السبب الرئيسي للعمي في جميع أنحاء العالم وفي المناطق الموبوءة بالتراخوما تكون التراخوما النشطة شائعة بين الأطفال في سن ما قبل المدرسة وذلك بنسب متفاوتة وتهدف الدراسة الحالية إلى تقدير مدي إنتشار التراخوما النشطة وعوامل الخطورة المرتبطة بها وذلك بين الأطفال في سن ما قبل المدرسة بمنطقة ديمبيا بشمال غرب إثيوبيا.
الأساليب: تم إجراء دراسة إستقصائية تداخلية على مستوي المجتمع المحلي بين الأطفال في سن ما قبل المدرسة بشمال غرب إثيوبيا وتم إستخدام عينات عشوائية متعددة المراحل لاختيار عدد 695 طفل وأخضع كل طفل لفحص عيني من قبل اختصاصيون طبيون مدربون في فحص البصر وذلك لتقييم إنتشار التراخوما النشطة بين الأطفال وأجريت مقابله شخصية باستخدام استبيان تم تنظيمه واختباره مسبقا وذلك لجمع البيانات عن عوامل الخطر المحتملة وقد رتبت حالات التراخوما بناءا علي تصنيف مبسط معد من قبل منظمه الصحة العالمية واجريت جميع التحليلات الإحصائية باستخدام النسخة رقم 20 من برنامج الحزمة الإحصائية للعلوم الاجتماعية مع إستخدام نسبة الأرجحية المعدلة (aORs) مع 95 ٪ من فترات الثقة لتحديد العوامل المرتبطة بالتراخوما النشطة.
النتائج: كانت التراخوما النشطة منتشرة بنسبة 18٪ في ال 681 طفل الذين تم دراستهم في مرحلة ما قبل الدراسة (من الصف الأول للصف الرابع) مع 95 ٪ من فترات الثقة تتراوح ما بين 15.4٪ و21.1٪ وكان الأطفال الذين لديهم وجوه نظيفة (غياب الإفرازات الأنفية والعينية) أقل فرصا لوجود التراخوما النشطة (بقيمة aOR تساوي 0.55 مع 95 ٪ من فترات الثقة تتراوح ما بين 0.37 و 0.82٪) وزادت احتمالات وجود التراخوما النشطة مع زيادة مسافة الوصول لمصدر المياه (بقيمة aOR تساوي 0.51 مع 95 ٪ من فترات الثقة تتراوح ما بين 0.33 و 0.78٪) وبالمثل فان الاستخدام الفقير او عدم استخدام حاويات للنفايات السائلة في المنزل الذي يعيش فيه الطفل كان مرتبطا بفرص متزايدة لوجود التراخوما النشطة (بقيمة aOR تساوي 3.83 مع 95 ٪ من فترات الثقة تتراوح ما بين 1.26 و 11.61٪).
الإستنتاج: تبين من الدراسة الحالية أن إنتشار التراخوما النشطة بين الأطفال في سن ما قبل المدرسة مرتفع ويحتاج إلى تدخلات خاصه تركز على تثقيف الأسر بشأن الغسيل الجيد للوجه والتخلص من الفضلات السائلة وتحسين الإمداد بالمياه النظيفة بالقرب من أماكن المعيشة.

Translated from English version into Arabic by Mohamed Habib

埃塞俄比亚西北部 Dembia 区学龄前儿童活动性沙眼的患病率及决定因素

Ayanaw Tsega Ferede, Abel Fekadu Dadi, Amare Tariku, Akilew Awoke Adane

摘要
引言：沙眼是由沙眼衣原体引起的传染性眼病，后者是全球主要的致盲性病原体。在沙眼流行地区，活动性沙眼在学龄前儿童很普遍，虽然具体流行程度不一。本研究旨在了解埃塞俄比亚西北部 Dembia 区学龄前儿童活动性沙眼的患病率及危险因素。
方法：在埃塞俄比亚西北部学龄前儿童开展了一项基于社区的横断面调查。采用多阶段随机抽样抽取了 695 个对象。经培训的临床眼科医生对每一个儿童进行视觉检查以评估是否患有活动性沙眼。采用经过预实验的、结构化问卷通过面对面的访谈收集潜在的危险因素。沙眼病例按照 WHO 简化评级系统进行分级。采用 SPSS 软件（20 版本）进行统计分析。采用调整的比值比（a*OR*s）及 95%可信区间（*CI*s）鉴别活动性沙眼的危险因素。
结果：在 681 名调查的 1-4 年级的学龄前儿童中，18%（95% *CI*：15.4% – 21.1%）患有活动性沙眼。面部清洁（无鼻部和眼部排泄物）的儿童患有活动性沙眼的几率较低（a*OR* = 0.55，95% *CI*：0.37 – 0.82）。与取水点距离较近，患有活动性沙眼几率降低（a*OR* = 0.51，95% *CI*：0.33 – 0.78 ）。类似地，家庭液体垃圾不处理或处理不足导致活动性沙眼患病几率增加（a*OR* = 3.83，95% *CI*：1.26 – 11.61）。
结论：这些学龄前儿童活动性沙眼患病率较高，因此需要针对家庭教育的专项干预措施，内容包括合理的面部清洗、液体垃圾处理和改善安全饮用水供应。

Translated from English version into Chinese by Men-Bao Qian, through

CHINESE CENTER FOR DISEASE CONTROL AND PREVENTION
NATIONAL INSTITUTE OF PARASITIC DISEASES

Prévalence et déterminants du trachome actif parmi les enfants d'âge préscolaire dans le district de Dembia au Nord-Ouest de l'Éthiopie

Ayanaw Tsega Ferede, Abel Fekadu Dadi, Amare Tariku, Akilew Awoke Adane

Résumé
Contexte: Le trachome est une maladie oculaire infectieuse causée par *Chlamydia trachomatis*, qui est la principale cause infectieuse de cécité dans le monde. Dans les zones où le trachome est endémique, le trachome actif dit inflammatoire est fréquent chez des enfants d'âge préscolaire, avec divers degrés de manifestation. Cette étude visait à estimer la prévalence du trachome actif et les facteurs de risque associés parmi les enfants d'âge préscolaire dans le district de Dembia au Nord-Ouest de l'Éthiopie.
Méthodes: Une étude transversale a été réalisée parmi les enfants d'âge préscolaire du Nord-Ouest de l'Éthiopie. Un échantillonnage aléatoire systématique à différentes phases a été fait pour sélectionner 695 personnes. Des optométristes cliniciens qualifiés ont soumis chaque enfant à un examen oculaire et évalué la présence du trachome actif. Un interrogatoire direct avec un questionnaire prétesté et structuré a été mené afin de recueillir des données sur les facteurs de risque possibles. Les cas de trachome ont été classés suivant le barème de classification simplifiée de l'Organisation Mondiale de la Santé (OMS). Toute analyse statistique a été effectuée en utilisant la version 20 du logiciel SPSS. Les quotients de probabilités ajustés [a*OR*s] avec des intervalles de confiance [*CIs*] à 95% ont été utilisés afin de déterminer les facteurs associés au trachome actif.
Résultats: Des 681 enfants en âge préscolaire (niveau 1 – 4) examinés, 18% (95% [*CIs*]: 15,4% – 21,1%) étaient atteints du trachome actif. Les enfants à visages dit ‘propres’ (absence d’écoulements nasaux et oculaires) avaient une faible probabilité d'être atteints du trachome actif [a*OR* = 0.55, 95% *CI*: 0.37 – 0.82]. Les probabilités d'avoir le trachome actif se sont accrues avec une augmentation de la distance à un point d'eau [a*OR* = 0.51, 95% *CI*: 0.33 – 0.78]. De même, aucune ou mauvaise pratique d’évacuation des déchets liquides des enfants dans les ménages a été associée au risque accru d'avoir le trachome actif [a*OR* = 3.83, 95% *CI*: 1.26 – 11.61].
Conclusion: La prévalence du trachome actif chez ces enfants d'âge préscolaire était élevée et il y a une nécessité d’interventions spéciales pouvant mettre l'accent sur l'éducation dans les familles et de façon adéquate sur le lavage de visage, l’élimination des déchets liquides, et l'amélioration de l'approvisionnement en eau potable près des ménages.

Translated from English version into French by Kokouvi Kassegne

Распространенность и детерминанты трахомой среди детей в дошкольных возрастах в районе Dembia на северо-западе Эфиопии

Ayanaw Tsega Ferede, Abel Fekadu Dadi, Amare Tariku, Akilew Awoke Adane

Аннотация
Введение: Трахома, это инфекционное заболевание глаз, вызываемое *Хламидией трахоматис*, которая является ведущей инфекционной причиной слепоты во всем мире. В некоторых районах трахома является эндемическим заболеванием, активная трахома распространяется среди детей в дошкольных возрастах с разной величины. Целью данного исследования было оценить распространенность трахомой и связанные факторы риска среди детей в дошкольных возрастах в районе Dembia на северо-западе Эфиопии.
Методы: Общинный кросс-секционный опрос проводился среди детей в дошкольных возрастах на северо-западе Эфиопии. Многоступенчатая систематическая случайная выборка используется для выбора 695 субъектов. Обучение клинических оптометристов подвергают каждого ребенка к офтальмологу и оценили наличии активной трахомы. Лицом

к лицу интервью, используя предварительное тестирование и структурированное анкетирование, чтобы собирать данные о возможных факторах риска. Случая трахомы были классифицированы по итогам Всемирной организации здравоохранения упрощенной схемы классификации. Весь статистический анализ проводился с помощью программного пакета SPSS версии 20. Скорректированные отношения шансов (a*OR*s) с 95% доверительными интервалами (*CIs*) были использованы для выявления факторов, связанных с трахомой.
Результаты: Из 681 детей, которые в дошкольных возрастах (класс 1 – 4), 18% (95% *CI*: 15.4% – 21.1%) имели заболеваемости трахомой. Дети, которые моются (отсутствие носовых и глазных разрядов) имели меньше шансов получить трахому [a*OR* = 0.55, 95% *CI*: 0.37 – 0.82]. Шансы, чтобы заболеть трахомой, увеличивается с увеличением расстояния до точки воды [a*OR* = 0.51, 95% *CI*: 0.33 – 0.78]. Аналогичным образом, отсутствие или плохое утилизации жидких отходов в доме ребенка было связано с увеличением шансов трахомой[a*OR* = 3.83, 95% *CI*: 1.26 – 11.61].
Выводы: Заболеваемость трахомы в эти дошкольники оказалась высокой и требует специальных вмешательств, направленных на воспитание семьей, правильное умывание, утилизацию жидких отходов, а также улучшение снабжения питьевой водой возле домовладений.

Translated from English version into Russian by Hao-Qi Zhang

Prevalencia y determinantes del tracoma activo entre niños en edad preescolar en el distrito de dembia, noroeste de Etiopía

Ayanaw Tsega Ferede, Abel Fekadu Dadi, Amare Tariku, Akilew Awoke Adane

Abstracto
Trasfondo: Tracoma es una enfermedad infecciosa ocular causada por *Chlamydia trachomatis*, la cual es la principal causa infecciosa de ceguera a nivel mundial. En las áreas donde el tracoma es endémico, el tracoma activo es común en niños de edad preescolar, con magnitud variante. Este estudio tuvo el objetivo de estimar la prevalencia del tracoma activo y sus factores de riesgo entre los niños de edad preescolar en el distrito de Dembia, al noreste de Etiopía.
Métodos: Se realizó una encuesta transversal de base comunitaria entre niños en edad preescolar del noroeste de Etiopía. Se utilizó muestreo aleatorio sistemático de múltiples etapas para seleccionar 695 sujetos. Optometristas clínicos entrenados sometieron a cada niño a un examen ocular y evaluaron la presencia de tracoma activo. Se realizaron entrevistas cara a cara utilizando un cuestionario preestablecido y estructurado para recopilar datos sobre posibles factores de riesgo. Los casos de tracoma se clasificaron siguiendo un esquema simplificado de clasificación de la Organización Mundial de la Salud. Todos los análisis estadísticos se realizaron utilizando la versión 20 del programa SPSS. Se utilizaron relaciones de probabilidad ajustadas (a*ORs*, por sus siglas en inglés) con intervalos de confianza del 95% (IC) para identificar factores asociados con el tracoma activo.
Resultados: De los 681 niños en edad preescolar (grado 1 - 4) estudiados, el 18% (IC del 95%: 15.4% - 21.1%) tenía una prevalencia de tracoma activo. Los niños que tenían caras limpias (ausencia de descargas nasales y oculares) tenían una menor probabilidad de tener tracoma activo [a*OR* = 0.55; IC del 95%: 0.37 – 0.82]. Las probabilidades de tener tracoma activo aumentaron con un aumento en la distancia a un punto de agua [a*OR* = 0.51; IC del 95%: 0.33 – 0.78]. Del mismo modo, la escasa o nula utilización de la disposición de desechos líquidos en el hogar del niño se asoció con una mayor probabilidad de tener tracoma activo [a*OR* = 3.83; IC del 95%: 1.26-11.61].
Conclusión: Se encontró que la prevalencia del tracoma activo en estos niños en edad preescolar es alta y necesita de intervenciones especiales que se enfoquen en educar a las familias sobre el lavado adecuado de la cara, la eliminación de desechos líquidos y mejorar el suministro de agua potable cerca de los hogares.

Translated from English version into French by Laura C Vicente Rodriguez
